# Protein kinase D1 regulates metabolic switch in pancreatic cancer via modulation of mTORC1

**DOI:** 10.1038/s41416-019-0629-9

**Published:** 2019-12-10

**Authors:** Sonam Kumari, Sheema Khan, Radhika Sekhri, Hassan Mandil, Stephen Behrman, Murali M. Yallapu, Subhash C. Chauhan, Meena Jaggi

**Affiliations:** 1 Department of Pharmaceutical Sciences and Center for Cancer Research, University of Tennessee Health Science Center, Memphis, TN USA; 2 Department of Immunology and Microbiology, University of Texas Rio Grande Valley, McAllen, TX USA; 3 Department of Pathology, University of Tennessee Health Science Center, Memphis, TN USA; 4 Department of Surgery, University of Tennessee Health Science Center, Memphis, TN USA

**Keywords:** Cancer metabolism, Cancer microenvironment

## Abstract

**Background:**

Protein kinase D1 (PKD1) is a serine–threonine kinase that regulates various functions within the cell. Herein, we report the significance of PKD1 expression in glucose metabolism resulting in pancreatic cancer (PanCa) progression and chemo-resistance.

**Methods:**

PKD1 expression in PanCa was investigated by using immunohistochemistry. Functional and metabolic assays were utilised to analyse the effect of PKD1 expression/knockdown on associated cellular/molecular changes.

**Results:**

PKD1 expression was detected in human pancreatic intraepithelial neoplasia lesions (MCS = 12.9; *P* < 0.0001) and pancreatic ductal adenocarcinoma samples (MCS = 15, *P* < 0.0001) as compared with faint or no expression in normal pancreatic tissues (MCS = 1.54; *P* < 0.0001). Our results determine that PKD1 enhances glucose metabolism in PanCa cells, by triggering enhanced tumorigenesis and chemo-resistance. We demonstrate that mTORC1 activation by PKD1 regulates metabolic alterations in PanCa cells. siRNA knockdown of Raptor or treatment with rapamycin inhibited PKD1-accelerated lactate production as well as glucose consumption in cells, which confirms the association of mTORC1 with PKD1-induced metabolic alterations.

**Conclusion:**

This study suggests a novel role of PKD1 as a key modulator of the glucose metabolism in PanCa cells accelerating tumorigenesis and chemo-resistance. The remodelling of PKD1-dysregulated glucose metabolism can be achieved by regulation of mTORC1 for development of novel therapeutic strategies.

## Background

Pancreatic cancer (PanCa) is one of the deadliest diseases, and the third most common cause of cancer-related deaths in the United States.^1^ The lack of early detection, chemo-resistance and increased metastasis contributes to the highly aggressive behaviour of this disease and poor survival rate.^1^ Therefore, it is essential to investigate and comprehend mechanisms that lead to the tumour aggressiveness and chemo-resistance in PanCa. One such unique characteristic physiology of pancreatic tumours includes a hostile and tumour microenvironment, which favours biochemical and metabolic adaptations to facilitate pancreatic tumour growth and metastasis. The metabolic alterations enable cells to satisfy their bioenergetic needs by adapting to the changing environments for survival, longevity and availability of nutritional requirements.^2^ Cellular signalling empowers cancer cells to acquire and process nutrients in a manner favourable to proliferation than production of ATP.^3^ Through aerobic glycolysis, cancer cells produce energy by converting glucose into lactate, in the presence of oxygen, which results in enhanced synthesis of lipids and nucleotides and stimulates growth and invasion. Herein, we postulate that the identification and strategic targeting of molecules that regulate altered glucose metabolism can help in the effective maintenance of the disease and development of therapeutics.

Protein kinase D1 (PKD1), a serine–threonine kinase, is an important modulator of several kinase signal transduction pathways.^4^ PKD1 belongs to the PKD family and is the most studied protein  kinase among the three family members (PKD1, PKD2 and PKD3).^4^ Previous reports have suggested that PKD1 is involved in pancreatic cancer pathogenesis,^5,6^ but the underlying signalling mechanisms are largely unknown. Studies have suggested the role of PKD1 in increasing pancreatic cancer proliferation and growth rate through stimulation of NFκB, an important transcription factor involved in various cellular mechanisms.^7^ PKD1 has also been reported to be involved in the Notch pathway, being downstream of KRAS, leading to pancreatic neoplasia.^5^ PKD1 expression contributes to formation of preneoplastic lesions in a KRAS^12D^-inducible transgenic model, suggesting an essential role of PKD1 in the initiation and progression of PDAC.^8^ However, there are no data available correlating PKD1 expression in human pancreatic intraepithelial neoplasia lesions (PanINs), which are early pancreatic cancer lesions. Although PKD1 being upregulated in acinar cells is known to regulate the duct formation leading to pancreatic cancer initiation and growth,^8^ only little is known of how the PKD1 regulates cancer development. In addition, PKD1 acts as one of the reactive oxygen species (ROS)- sensing molecule in the tumour microenvironment, increases oxidative stress, ultimately resulting in pancreatic cancer progression.^6^ Therefore, we were interested to further investigate the PKD1 expression during PDAC development by using human PDAC tissue samples and molecular changes influenced by PKD1 expression that may contribute to pancreatic tumorigenesis. Our objective was to understand the underlying molecular mechanisms involved in PKD1-induced aberrant glucose metabolism and enhanced aggressiveness of pancreatic cancer.

One of the most important pathways regulating metabolic processes is the mTOR (mammalian target of rapamycin) pathway.^9^ mTOR is a serine/threonine kinase that exists as two different complexes, mTORC1 and mTORC2. mTOR, in association with Raptor and mLST8 forms mTORC1, regulates protein translation, cell-cycle progression and metabolism.^2^ While mTOR in association with Rictor (rapamycin-insensitive companion of mTOR) and mSIN1 (stress-activated protein) forms mTORC2 and regulates cell survival/glucose metabolism and cytoskeletal remodelling.^9^ Rapamycin is known to inhibit mTORC1 activity and promotes β-oxidation for anabolic storage pathways.

Thus, in this study, we present the expression profile of PKD1 in human PanINs (early–late), PDAC and chronic pancreatitis tissues and determine its correlation with different tumour differentiation status (well–poorly). In addition, this study suggests a novel role of PKD1 in regulating glucose metabolism in pancreatic cancer, which drives pancreatic tumorigenesis, chemo-resistance and progression. This takes place in correlation with mTOR signalling, wherein mTORC1 predominantly regulates PKD1-induced metabolic changes. PKD1 is aberrantly expressed in PanCa. The idea was to identify novel mechanisms to develop newer therapeutic strategies for this exceptionally difficult cancer. By showing that PKD1-mediated reprogramming of glucose metabolism regulates the metabolic demands of malignant cells, we describe a novel mechanism that is governed by PKD1 expression in PDAC. Briefly, our study prospects PKD1 as a novel molecular target in pancreatic cancer for therapeutic intervention.

## Methods

### Cell culture

All pancreatic cells (normal; HPNE and cancerous; HPAF-II, Panc-1, Capan-1, MiaPaca, AsPC1 and BxPC3) used in this study were obtained from ATCC. Among these, HPAF-II/BxPC3 are low PKD1 expressing, while Panc-1/AsPC1/Capan-1/MiaPaca are determined high PKD1-expressing cells. All cells were maintained in DMEM/F12 or RPMI media containing 10% foetal bovine serum in an incubator at 37 °C temperature with 5% CO_2_. The normal human pancreatic cell line, HPNE, was maintained in DMEM media. The complete media, and the cell lines were maintained. The cells were frequently investigated for mycoplasma growth and contamination.

### Procurement and scoring of human pancreatic cancer tissues for analysis of PKD1 expression

Pancreatic cancer tissue microarray was procured from US Biomax (# BIC14011a SL160) with chronic pancreatitis, early PanINs, late PanINs and PDAC cores. Also, normal tissues were obtained from Baptist Memorial Hospital (BMH), Memphis, TN. The scoring of the PKD1 expression was performed as described earlier in detail.^10^ Stained slides were digitally scanned and analysed for PKD1 staining at the University of Tennessee Health Science Center (UTHSC) Pathology Department. Captured images were independently analysed and scored by three reviewers, who were kept blinded to the patient history. The intensity of immunoreactivity of PKD1 was graded on a 0–4 scale (0 for no staining, 1 for weak immunoreactivity, 2 for moderate immunoreactivity, 3 for strong immunoreactivity and 4 for very strong immunoreactivity). The percentage of cells positive for PKD1 immunoreactivity within the tumour and normal tissue sections were scored as follows: 0–25% as 1, 26–50% as 2, 51–75% as 3 and 76–100% as 4. The mean composite score (MCS) was determined by calculating an average of the composite scores (intensity × percentage) of the respective samples in each category.

### Western blotting

The whole-cell lysates from pancreatic cancer cells were prepared and used for investigating the changes in protein expression through western blotting procedure, as described before.^11,12^ Briefly, 40 µg of protein was loaded per well to perform SDS PAGE followed by western blotting. The transfer of gels was done on PVDF (polyvinylidene difluoride) membrane and membranes were blocked in either 5% nonfat dairy milk or 5% BSA (bovine serum albumin) for 1 h followed by incubation with the primary antibodies overnight at 4 °C. The primary antibodies with the following catalogue numbers were procured from Cell Signaling Technology: HIF-1α (3716), Glut-1 (12939), Ras (3965), KRAS-12D (14429), eIF4e (9742S), peIF4e (9741S), 4EBP1 (9452S), p4EBP1 (2855S), mTOR (4517S), and p-mTOR (5536S). PKD1 antibody (sc-639) was procured from Santa Cruz Biotechnology. β-actin antibody was obtained from Sigma (A2228). The rabbit (4011) and mouse (4021) secondary antibodies were purchased from Promega company.

### Immunofluorescence

Immunofluorescence was conducted as described earlier.^13^ Briefly, cells were fixed, permeabilised and incubated overnight with primary antibodies. The following day, the cells were incubated with the respective secondary antibodies for 1 h, washed and mounted. Further, the images were taken under a confocal microscope at ×400 magnification (Zeiss 710, Germany).

### Immunohistochemistry

Pancreatic cancer human tissue microarrays were obtained from Baptist Memorial Hospital, Memphis, and were immunostained by using antigen-retrieval method, as described earlier^10^ by using a commercially available Biocare kit from Biocare Medical, CA, USA. Briefly, the tissue slides were deparaffinised, rehydrated, peroxidised and antigen retrieved. Further, the tissues were incubated with primary antibodies overnight followed by addition of secondary MACH4 Universal HRP mouse polymer (#M4U534H) followed by staining with 3-diaminobenzidine (DAB) chromogen (#DB801) and counterstaining with haematoxylin.

### Transfection of cells

Pancreatic cancer cells having low PKD1 expression (HPAF-II and BxPC3) were incubated in serum-free OPTI-MEM media overnight, and transiently transfected with PKD1 expression plasmid and control pEGFP by using Lipofectamine 2000 reagent. Following 6 h of transfection, 10% serum-containing regular media was added to the cells after removing the old media. The transfection procedure was terminated after 48 h, and the cells were used for further experiments.

### Lactate assay/glucose assay

Lactate and glucose assays were performed by utilizing commercially available kits from Cayman Chemicals (lactate assay kit: catalogue No. 600450 and glucose assay kit: catalogue No. 10009582). Cells were seeded as per the manufacturer’s instructions in the kit, and media of cells was used to determine the amount of lactate production and glucose consumption upon termination of the experiment at 24 and 48 h. Further, the absorbance was recorded at the indicated wavelength, and readings were analysed/calculated.

### Cell migration assay

The cell migration assay was conducted by using Boyden chamber plate.^11,14,15^ In total, 50,000 pancreatic cancer cells were seeded on the top chamber of the 96-well plate in a serum-free media, and 250 µl of serum-containing media was added to the lower chamber. l-Lactate (2 mM) or 2-deoxyglucose (2DG: 10 mM) were added to the cells, which was followed by incubation for 18 h at 37 °C. Thereafter, the cells were fixed with 4% paraformaldehyde, stained with crystal violet and washed with water. The images of cells were captured after complete drying of the plate.

### Cell invasion assay

Cell invasion assay was performed by using Matrigel Invasion Chambers from BD Biosciences.^14^ In total, 25,000 cells were seeded in the chambers containing media without serum in the upper chamber and complete media (10% FBS) in the lower one. l-lactate (2 mM) or 2-deoxyglucose (10 mM) were added to the cells, which was followed by incubation for 24 h at 37 °C. Further, the cells were fixed by methanol and stained by using crystal violet. The plates were washed with water and left to dry, which was followed by capturing images.

### MTT assay/cell proliferation assay

MTT assay was performed in PKD1-expressing and null cells to determine the effect of PKD1 in cell proliferation as described before.^11^ Following 48 h, 20 µl of MTT reagent was added to the cells. Then, 2 h after the addition of this reagent, 100 µl of dimethyl-sulfoxide (DMSO) solution was added on top of the cells, and the plate was subjected to vigorous shaking for another 10 min. Next, the absorbance was read at a wavelength of 570 nm, the cell viability was calculated and the graph was plotted.

### Colony formation assay

The pancreatic cancer cells were seeded in a 12-well plate (250 cells/well). The cells were subjected to both normoxic and hypoxic conditions at the same time. For hypoxia experiments, the cells were cultured at 37 °C with 5% CO_2_, 94% N_2_ and 1% O_2_ in a multi-gas incubator. The experiment was terminated after 7 days with  PBS wash, fixed with 100% methanol and stained for 10 min with crystal violet. The stain was carefully removed by thoroughly washing with water, which was followed by air drying the plate and capturing images by using a microscope. The colonies (>50 cells) were counted manually and plotted as described earlier.^16^

### Quantitative polymerase chain reaction (qPCR)

RNA samples were isolated from various pancreatic cancer cells by using Qiagen kit. The reaction was conducted as described earlier.^11^ The concentration of samples was determined, and 2 µg of RNA was reverse transcribed. The expression of genes (PKD1, HIF-1α and Glut-1) was determined by precise primers through real-time PCR experiment. GAPDH served as an internal control.

### Statistical analysis

The significance of the data in this paper was determined through Student’s *t* test, and *p*-values below 0.05 were set to be statistically significant.

## Results

### PKD1 expression is upregulated in human pancreatic cancer tissues and cells

**Fig. 1:**
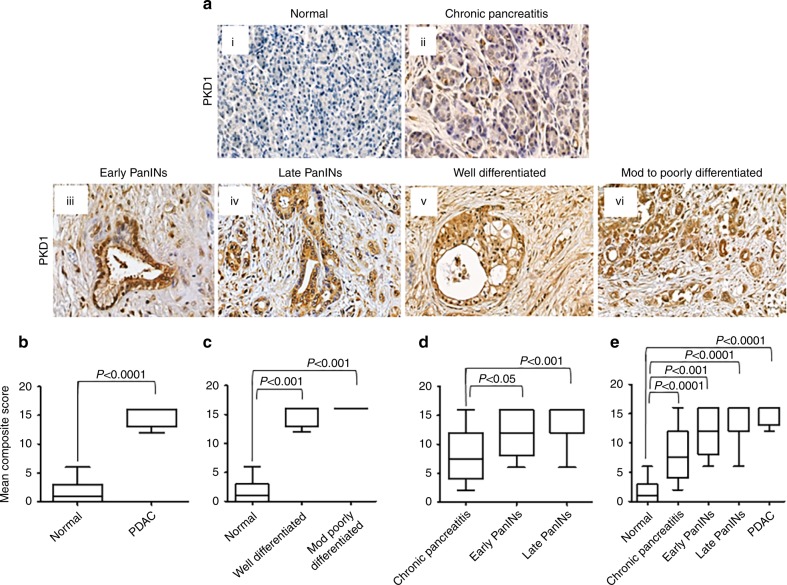
PKD1 is highly expressed in human pancreatic cancer tissues. **a** Immunohistochemistry showing the expression of PKD1 protein in normal (i), chronic pancreatitis (ii), early–late PanINs (iii and iv) and well–mod–poorly differentiated pancreatic cancer human tissues (v and vi). **b** Boxplot representing quantification and depiction of mean composite score in the overall normal and PDAC tissues for PKD1. **c** Mean composite score in normal pancreas and different grades of pancreatic cancer tissues. **d**, **e** Comparison of mean composite score of normal and malignant pancreatic tissues (magnification ×400)

The expression profile of PKD1 in human pancreatic cancer tissues and normal samples (*N* = 45) was investigated by using immunostaining of PKD1 by immunohistochemistry. The samples included early pancreatic intraepithelial neoplasia (PanINs), late PanINs, pancreatic ductal adenocarcinoma (PDAC), normal pancreas and chronic pancreatitis. We evaluated the staining scores in Mean Composite Score (MCS) by taking into account of the average staining pattern. The MCS calculated for all tissues was obtained by multiplying percentage intensity and the percentage of tissue staining. Normal pancreatic tissues and chronic pancreatitis tissues showed none or very faint PKD1 expression (Fig. 1a i–ii). PKD1 expression was observed as early as in PanIN lesion formation (Fig. 1a iiii–iv), whereas all of the stained PDAC tissues showed moderate-to-high PKD1 expression (Fig. 1a v–vi). Overall, PKD1 expression was significantly higher (MCS: 15, *P* < 0.0001) in PDAC compared with normal pancreatic ductal epithelium (Fig. 1a, b). PKD1 expression was predominantly expressed all over the malignant area of the pancreatic cancer tissues when compared with respect to grade (Fig. 1c), PanIN lesions (Fig. 1d) and overall comparisons (Fig. 1e). Our results clearly indicate that PKD1 expression effectively differentiated PDAC from healthy controls and chronic pancreatitis. These results further suggest that PKD1 is overexpressed in pancreatic cancers and might have an oncogenic role in the malignant transformation of pancreatic cells. These results were in concordance with the information available online in Oncomine database within two different datasets (Segara and Badea).^17,18^ PKD1 was observed to be highly expressed in pancreatic cancer tissues as compared with normal/non-malignant pancreatic tissues (Supplementary Fig. S1).

**Fig. 2:**
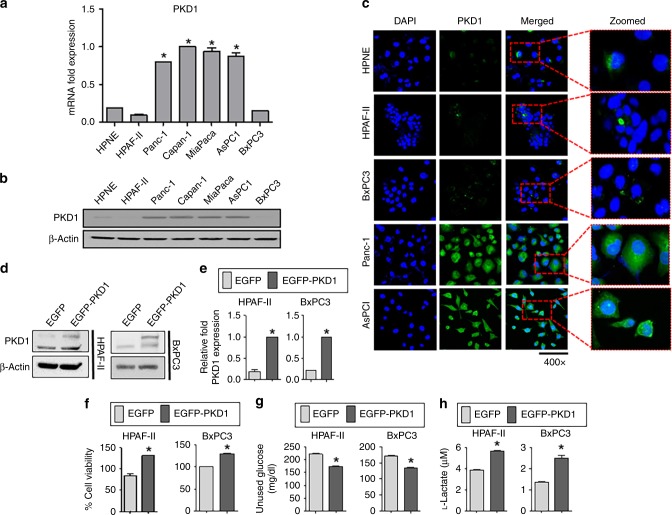
Differential expression of PKD1 in various pancreatic cancer cell lines and effect on glucose metabolism. **a** Real-time PCR depicting the expression level of PKD1 in a panel of pancreatic cancer cell lines. HPNE is a normal pancreatic cell line. **b** Western blot showing the expression level of PKD1 in different pancreatic cancer cell lines. **c** Confocal immunofluorescence images showing the expression of PKD1 in pancreatic cancer cells. Cells were fixed, stained and analysed by confocal microscopy. DAPI was used as a counterstain for the nucleus. Zoomed images in the inset show the cytoplasmic expression of PKD1 protein. **d** Pancreatic cancer cells (HPAF-II and BxPC3) were transfected with control EGFP and EGFP-PKD1 plasmid, and the overexpression of PKD1 was confirmed by using immunoblotting and (**e**) real-time PCR. **f** Cell proliferation assay for 48 h, (**g**) glucose and (**h**) lactate assays performed in HPAF-II and BxPC3 cells after PKD1 transfection. Cell culture media was collected after 48 h to measure the amount of unused glucose and l-lactate concentration by using glucose and lactate assay kits. *P*-values are denoted by *P < 0.05

We also investigated the expression level of PKD1 in a normal pancreatic epithelial cell line (HPNE) and six pancreatic cancer cells, which includes HPAF-II, Panc-1, Capan-1, MiaPaca, AsPC1 and BxPC3 cells. Except HPAF-II and BxPC3, which showed less expression of PKD1, most pancreatic cancer cells had high expression of PKD1 at both mRNA (PCR, Fig. 2a) and protein levels (immunoblotting and confocal immunofluorescence, Fig. 2b, c). We observed increased cytoplasmic expression of PKD1 in cells.

### PKD1 contributes to tumorigenic characteristics via glucose modulation

**Fig. 3:**
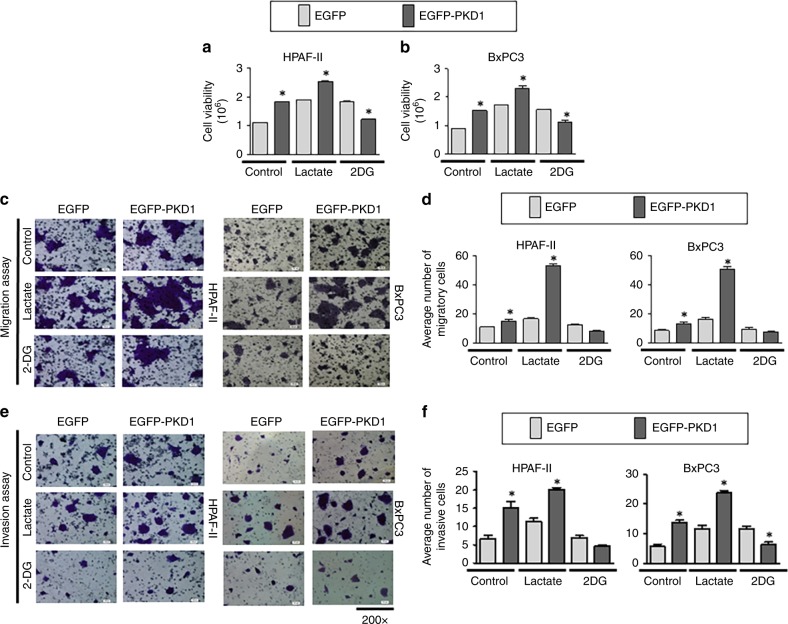
PKD1 modulates glucose metabolism and enhances proliferation, invasion and migration in pancreatic cancer cells. HPAF-II and BxPC3 cells were treated with l-lactate (2 µM) and 2-deoxyglucose (2DG, 10 µM). **a**, **b** MTT reagent was added to the cells, and absorbance was recorded following 48 h. **c**–**f** Boyden chamber migration and Matrigel invasion assays were performed by staining cells with crystal violet following 18 and 24 h, respectively. The migratory and invasive cells were quantified and are represented by the plotted graphs. All images were captured at ×200. *P*-values are denoted by *P < 0.05

PKD1 overexpression or activation has been reported to facilitate survival and proliferation of pancreatic cancer cells.^19^ In addition, PKD1 is involved in transdifferentiation of pancreatic acinar cells to a duct-like, pluripotent progenitor cell type (ADM, acinar-to-ductal metaplasia), leading to the initiation of pancreatic cancer.^8,20^ Although studies suggest the oncogenic role of PKD1 in pancreatic cancer, the precise mechanisms directly influencing the PKD1-induced tumorigenic characteristics are not elucidated. These studies are essential to develop strategies to control malignant transformation as well as increased tumour growth in pancreatic cancer. To investigate the underlying molecular mechanisms, we generated PKD1-overexpressing HPAF-II and BxPC3 cells (having low PKD1) by using transient transfection. The transient transfection was performed to observe the abrupt molecular changes in the cell upon altering the PKD1 expression. We observed an increase in the protein and mRNA levels of PKD1 expression in both the transfected cells, HPAF-II and BxPC3 by using immunoblotting and PCR, respectively (Fig. 2d, e). We observed an increase in the percentage of cell viability on overexpression of PKD1 in both cells, HPAF-II and BxPC3 (Fig. 2f; Supplementary Fig. S2A, B). In order to investigate the role of PKD1 on glucose modulation in PanCa cells, glucose and lactate assays were performed in PKD1-overexpressing cells, HPAF-II and BxPC3. We observed significantly (*p* < 0.05) higher upregulation of glucose consumption and l-lactate production in PKD1-overexpressing cells as compared with the control cells having basal levels of PKD1 (Fig. 2g, h). Therefore, these results indicate that PKD1 contributes to altered glucose metabolism of pancreatic cancer cells. Our results further demonstrated increased migration (Fig. 3a, b) and invasive (Fig. 3c, d) potential of HPAF-II and BxPC3 cells with PKD1 overexpression as compared with the control cells with basal levels of PKD1. We further investigated if the changes in glucose metabolism alter the enhanced tumorigenic characteristics of the cells on PKD1 overexpression. Our results demonstrate that the proliferation (Fig. 3a, b), migration (Fig. 3c, d) and invasive (Fig. 3e, f) potential of PKD1-overexpressing cells can be reduced by inducing glucoprivic conditions by using 2-deoxyglucose or potentiated by using lactate (2 mM), an end product of aerobic glycolysis, in the cells. This further suggested the involvement of glucose-accelerated mechanisms acquired by PanCa cells on PKD1 overexpression that accompanies enhanced tumorigenesis.

### PKD1 leads to increased expression of signalling proteins that trigger enhanced glucose metabolism

**Fig. 4:**
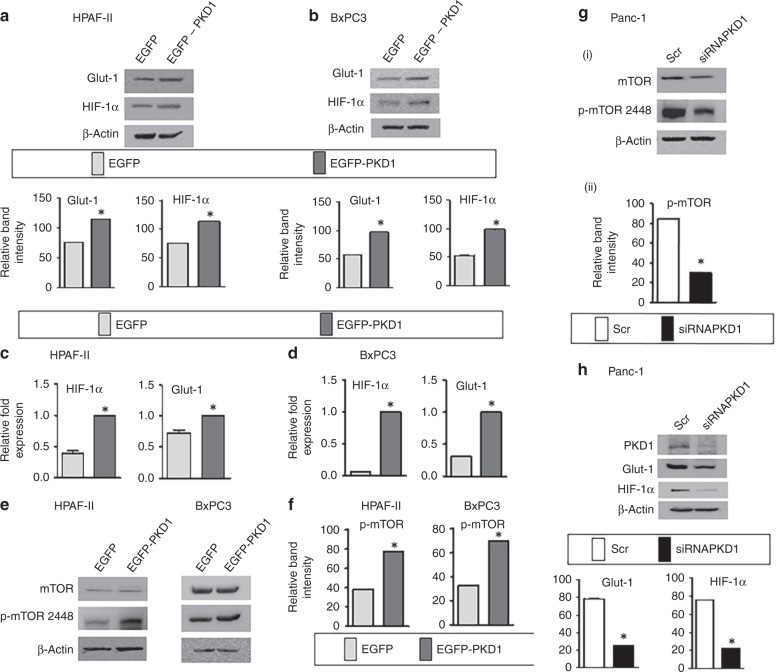
PKD1 modulates the expression of important genes and proteins involved in enhanced cancer cell metabolism. **a**, **b** Western blot showing the expression level of key proteins associated with glucose metabolism, Glut-1 and HIF-1α following overexpression of PKD1 in HPAF-II and BxPC3 cells and **c**, **d** changes in real-time expression of Glut-1 and HIF-1α by quantitative real-time PCR in HPAF-II and BxPC3 cells. **e** Immunoblotting depicting the expression of mTOR and p**-**mTOR-S2448 in pancreatic cancer cells following PKD1 overexpression and (**f**) bars representing relative quantification of p-mTOR band intensity. **g** i–ii Immunoblotting depicting the mTOR and p**-**mTOR-S2448 protein expression levels in Panc-1 cells upon silencing of PKD1 and bars representing the relative quantification of p-mTOR band intensity. **h** Silencing PKD1 in Panc-1 cells having high expression of PKD1. The lower panel represents the densitometry analysis of band intensity. *P*-values are denoted by *P < 0.05. The blots were re-probed multiple times with β-actin being used as a protein-loading control

Alteration of metabolic processes requires the interplay between a plethora of signalling events involving changes in the expression of proteins. Glucose transporter-1 or GLUT1 is an important protein that facilitates the transport of glucose across the plasma membranes of cancer cells.^21^ Our results demonstrate that PKD1 overexpression in PanCa cells, HPAF-II and BxPC3, results in an upregulation of protein as well as mRNA expression of GLUT1 (Fig. 4a–d). We also observed an increased protein (Fig. 4a, b) and mRNA expression (Fig. 4c, d) of hypoxia-inducible factor or HIF-1α, which has a role in metabolic switching and regulates glucose metabolism.^22^ Therefore, we were interested to understand the underlying molecular mechanisms that may be involved in metabolic alterations accompanied by PKD1. Since mTOR signalling is widely reported to regulate HIF‑1α protein synthesis, we particularly investigated mTOR pathway.^23^ mTOR is known as a master regulator of growth and metabolic state of cells in response to nutrients and extracellular stimuli. Interestingly, our results clearly demonstrated that PKD1 overexpression enhances the phosphorylation of mTOR (Fig. 4e, f). These results were confirmed upon silencing of endogenous PKD1 in Panc-1 cells (having high PKD1 expression) that inhibited the aforementioned events (Fig. 4g i and ii). In addition to the phosphorylation of mTOR, endogenous expression of GLUT1 and HIF-1α was observed to be reduced upon silencing endogenous PKD1 expression (Fig. 4h), which suggests that PKD1-induced metabolic changes may be influenced by mTOR phosphorylation. We next sought to investigate whether the hypoxic environment has any effect on PKD1-induced tumorigenic effects. BxPC3 and HPAF-II cells cultured in a hypoxic environment displayed high clonogenicity with formation of a greater number of colonies on PKD1 overexpression as compared with cells under normoxic environment (Supplementary Fig. S3).

### PKD1 modulates glucose metabolism via mTORC1 activation

**Fig. 5:**
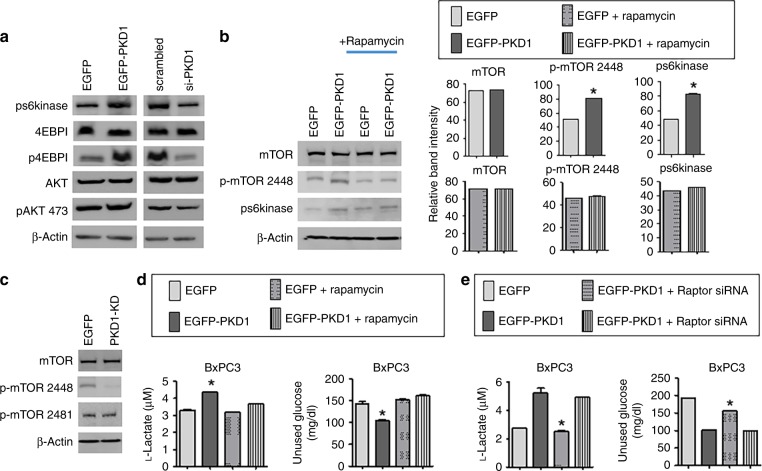
PKD1 increases glucose metabolism via mTORC1 activation. **a** Western blot to analyse the expression level of important proteins associated with mTORC1 and mTORC2 complex following overexpression and silencing of PKD1 in HPAF-II and Panc-1 cells, respectively. **b** Immunoblotting depicting the expression of p-mTOR-S2448 and ps6kinase, the major downstream effectors of mTORC1 in the presence and absence of rapamycin. The right panel represents the densitometry analysis of band intensity. **c** Immunoblotting showing the expression of phosphorylated p-mTOR 2448 and p-mTOR-S2481, the major phosphorylating domain of mTORC1 and C2, respectively, following transfection with PKD1 kinase dead domain. **d** Lactate and glucose assay in PKD1-expressing, BxPC3 cells in the presence and absence of rapamycin and **e** Raptor and Rictor siRNA. *P*-values are denoted as *P < 0.05. The blots were re-probed multiple times with β-actin being used as a protein-loading control

We set out to analyse whether PKD1-induced mTOR phosphorylation involves either mTORC1 or mTORC2 phosphorylation. mTORC1 is known to modulate HIF-1α, which stimulates glycolysis and glucose uptake. In order to elucidate the possible involvement of mTORC1, the major downstream effectors of mTORC1 are the ribosomal S6 kinase (S6K) and the inhibitory eIF4E-binding proteins (4E-BPs). mTORC1 activation phosphorylates S6K and 4E-BP, which phosphorylates/activates downstream proteins involved in initiation and elongation.^24^ We found that PKD1 overexpression in PanCa cells results in enhanced phosphorylation of both the effectors of mTORC1, pS6kinase and 4EBP1 (Fig. 5a). To our expectation, the silencing of endogenous PKD1 inhibited phosphorylation/activation of both the downstream effectors of mTORC1. In addition to increased expression of pS6kinase and 4EBP1, an upregulation of p-AKT, a major effector of mTORC2, was observed on PKD1 overexpression (Fig. 5a) suggesting the activation of both the complexes of mTOR, mTORC1 and mTORC2. Since mTORC1 is rapamycin sensitive, we investigated the effect of PKD1 overexpression in the presence of rapamycin on the phosphorylation of pS6kinase and mTOR-S2448, the major phosphorylating domain of mTORC1. We observed that rapamycin treatment prevented the increase in phosphorylation of both pS6kinase and mTOR-S2448, indicating that mTORC1 is the main effector of PKD1 (Fig. 5b). It is known that the kinase domain of PKD1 is involved in the activation/phosphorylation of proteins.^25^ Therefore, in order to investigate whether the kinase region is responsible for the increased activity/phosphorylation of mTOR, BxPC3 cells were transfected with the PKD1 kinase dead domain (PKD1-KD). Immunoblotting demonstrated inhibition in the phosphorylation of mTOR-S2448 but not mTOR-S2481 (the major phosphorylating domain of mTORC2) in cells transfected with PKD1-KD. These results indicate two important conclusions: PKD1 directly phosphorylates mTOR-S2448 through its kinase domain and mTORC1 being the downstream effector of PKD1 (Fig. 5c). We next asked whether mTORC1 is directly involved in PKD1-modulated glucose metabolism. For this, we investigated the changes in the lactate production and glucose consumption in PKD1- overexpressed cells in the presence or absence of rapamycin. Our results clearly show that the PKD1-expressing cells, in the presence of rapamycin, failed to increase lactate production and glucose consumption (Fig. 5d). These effects were confirmed by using siRNA-mediated knockdown of Raptor or Rictor, which are the determinants of the activation of mTORC1 and mTORC2, respectively,^26^ in the presence of PKD1 overexpression in cells. siRNA directed to Raptor but not Rictor, inhibited the lactate production and glucose consumption triggered by PKD1 (Supplementary Figs. S4 and 5E). No noticeable changes were observed upon silencing Rictor. As Raptor is an important component of mTORC1 complex,^26^ these results indicated an obvious role of the mTORC1 pathway in the aberrant metabolism induced by PKD1.

### PKD1 expression leads to drug resistance and its inhibition sensitises gemcitabine response

**Fig. 6:**
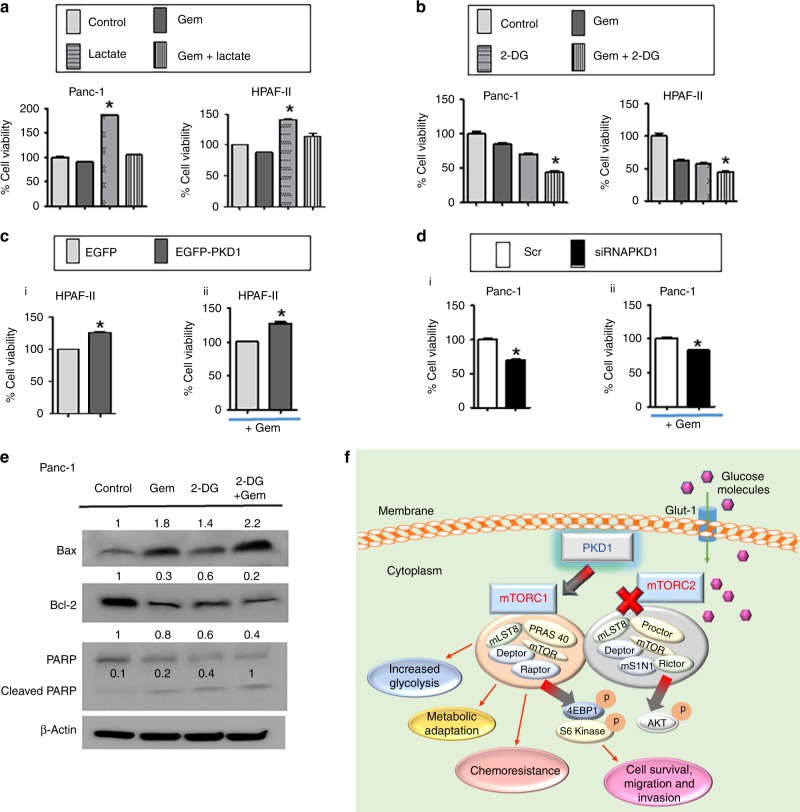
PKD1 modulation sensitises gemcitabine response in pancreatic cancer cells. **a** Effect of cell proliferation on treatment with gemcitabine following l-lactate and **b** 2DG supplementation. **c** i Effect of PKD1 overexpression on cell viability and **c** ii gemcitabine response in HPAF-II cells. **d** i Effect of PKD1 silencing on cell viability and **d** ii sensitization of gemcitabine. **e** Immunoblotting assay demonstrating the changes in the expression of apoptotic proteins following treatment with gemcitabine in the presence and absence of 2DG. *P*-values are denoted as *P < 0.05. **f** Schematic representation depicting the involvement of PKD1 that regulated the molecular signalling pathway that causes impaired glucose metabolism in pancreatic cancer. PKD1 acts as the main regulator of the glucose metabolic network through Glut-1 and mTORC1 activation, which in turn leads to increased metabolic adaptation in pancreatic cancer cells resulting in enhanced proliferation, invasion and chemo-resistance in pancreatic cancer cells. The blots were re-probed multiple times with β-actin being used as a protein-loading control

High glucose accelerates cell proliferation in pancreatic cancer cells and imparts chemo-resistance. Metabolic dysregulation is one of the most common and recognisable features of cancer. This facilitates the change in response to substrate availability due to metabolic demands of the cell during proliferation, growth and cell survival.^11^ Cancer/proliferating cells alter their ability to metabolise biomolecules to meet increased energy demands. It has been recognised that cancer cells increase glycolytic lactate production independent of oxygen availability.^27^ In addition to studies related to the role of PKD1 in glucose metabolism, we were interested to investigate its effect on anticancer therapy. Therefore, we wanted to investigate the effect of aberrant glucose metabolism on the outcome of drug treatment in PanCa cells, and whether response to drugs can be affected by excessive glucose metabolism. Therefore, upon treatment of cells with lactate (2 µM, 48 h), we observed an increase in the viability of the cells and resistance to gemcitabine treatment (Fig. 6a). Treatment of cells with 2DG resulted in decreased viability and restored gemcitabine sensitivity (Fig. 6b). In order to investigate the role of PKD1 in chemo-resistance, we analysed the effect of PKD1 overexpression on cell viability and gemcitabine resistance. As a result, we observed an enhanced cell viability on PKD1 overexpression (Fig. 6c i) and resistance to gemcitabine (Fig. 6c ii). Suppression of PKD1, by using siRNA-mediated knockdown, reduced the cell viability as well as gemcitabine resistance (Fig. 6d i and ii). In addition, an induction of apoptosis by gemcitabine was observed in the presence of 2DG as depicted by activation of apoptotic protein, Bax, cleavage of PARP and inhibition of antiapoptotic Bcl-2 (Fig. 6e). These outcomes indicate that expression of PKD1 in pancreatic cancer cells results in chemo-resistance, which attributes to the deregulated glucose metabolism, and its suppression sensitises gemcitabine in pancreatic cancer cells (Fig. 6f).

## Discussion

Excessive glucose metabolism is a significant characteristic feature of cancer. Pancreatic cancer cells require large amounts of glucose for their survival. It leads to the fast growth and proliferation of the cancer cells. Many signalling molecules are reported to play an essential role in the aberrant metabolic processes, but further studies are still required to develop effective treatment strategies targeting glucose metabolism.^28^ Several reports indicate an oncogenic role of the PKD1 protein in pancreatic cancer, but the mechanisms involved have not been sufficiently defined.^5,29^ Therefore, in this study, we report a novel role of PKD1 in rewiring of glucose metabolic network that drives favourable tumour microenvironment and oncogenic signalling pathways in pancreatic cancer cells. This study demonstrates the role of PKD1 in enhancing glucose metabolism of pancreatic cancer cells and defines the underlying molecular mechanisms. In addition, this study determines an important role of PKD1 in pancreatic cancer chemo-resistance.

In this study, we demonstrate that PKD1 is highly expressed in PanIN lesions and human PDAC as compared with less or no expression in normal pancreatic tissues. This is the first study to our knowledge, which presents the expression profile of PKD1 in human PDAC. The oncogenic role of PKD1 in PanCa is growing interest among the scientific community and several reports suggest its role in pancreatic tumorigenesis.^5,29,30^ Herein, we demonstrate a functional role of PKD1 in maintaining glucose metabolism of PanCa cells and illustrate signalling mechanisms that regulate the underlying molecular processes accompanying altered glucose metabolism. Glucose transporter-1 (GLUT1) mediates cellular transport of glucose required for anaerobic metabolism in cancer cells.^31^ Oncogenic K-Ras mutation, a signature event in pancreatic cancer, induces GLUT1 expression resulting in anabolic glucose metabolism.^32^ Recent studies suggest the association of KRAS mutations with the increased PRKD1 promoter.^8^ Although a precise molecular mechanism of how PKD1 induces GLUT1 expression, is not illustrated in this paper, we speculate a functional link between the activation of PKD1 with the oncogenic KRAS mutations induced by GLUT1 expression. Our studies suggest that PKD1 overexpression enhances glucose uptake by increasing expression of GLUT1 in PDAC cells. The increased GLUT1 expression enhances intracellular glucose concentration due to augmented GLUT1-dependent glucose uptake in cells, which accompanies extreme lactate production and glucose consumption. Our results determine that PKD1 overexpression leads to enhanced lactate production and glucose consumption while its knockdown reversed the effect. The results are similar with the supplementation of cells with lactate that potentiates the migration and invasion of PanCa cells. While under glucoprivic conditions, in the presence of 2DG (a glucose analogue that inhibits glycolysis),^27,33^ restores the PKD1- induced tumorigenic effects. These data suggest that PKD1 promotes tumorigenesis by modulating glucose metabolism and warrants further investigation for the underlying mechanisms. As PKD1 was also observed to affect the expression of hypoxia-inducible factor 1 (HIF-1), further investigations revealed hypoxic environment being conducive to increased effects on cell growth, as depicted by the ability of cells to colonize (Supplementary Fig. S3) and warranted further investigation. However, these results corroborated with recently published studies suggesting that protein kinase D1 regulates HIF-1α in the human tongue squamous cell carcinoma cells.^34^

mTOR is the conserved serine/threonine kinase that regulates the proliferation and metabolism in cells. mTOR exists in two distinct complexes with different protein components and downstream substrates: mTOR complex C1 and C2.^35^ mTORC1 stimulates HIF-1α by regulating various enzymes for glucose metabolism, including glucose transporters.^36^ This study demonstrates that PKD1 phosphorylates/activates mTORC1 in pancreatic cancer cells, as was depicted by the increased activation/phosphorylation of its main effectors, pS6K and 4EBP1 on PKD1 overexpression (Fig. 5). In addition, the kinase dead mutant of PKD1 did not enhance the phosphorylation/activation of the major phosphorylating domain of mTORC1, mTOR-S2448. The observed reduction in the phosphorylation of mTOR-S2448 in cells expressing kinase mutant of PKD1 can be well explained with the fact of a new phosphorylated protein not being produced, while the accumulated protein being degraded with time (Fig. 5c). These results indicate that PKD1 activity is necessary for mTORC1 activation. mTORC1 inactivation by rapamycin is known to be critical for glucose metabolism.^37,33^ Rapamycin binds to the FK506-binding protein of 12 kDa (FKBP12) and inhibits the activity of mTORC1.^38^ Our findings demonstrate that mTORC1 activation by PKD1 regulates metabolic alterations in pancreatic cancer cells. These results were quite convincing as the treatment of rapamycin inhibited accelerated lactate production as well as glucose consumption due to PKD1 overexpression in cancer cells. In addition, the significance of mTORC1 activation in PKD1- induced metabolic alterations was established upon knockdown of Raptor, the main determinant of the activation of mTORC1, which ultimately inhibited the metabolic changes associated with PKD1. Since no significant alterations of metabolic changes were detected upon silencing Rictor, these results further confirm the association of mTORC1 with PKD1-induced metabolic alterations. In other words, it can be stated that the promotion of glycolysis by PKD1 can be regulated by mTORC1 suppression and this concept can be utilised to develop novel therapeutic strategies.

Another major finding in this study demonstrates the role of PKD1 in chemo-resistance. The results presented in this study suggesting that PKD1 induces chemo-resistance due to aberrant glucose metabolism, are significant. In addition, we demonstrate that altered glucose metabolism interferes with the sensitivity of the drug and its outcomes in PanCa cells. Our data suggest that PanCa cells become resistant to chemotherapeutic drugs in the presence of enhanced glucose, which can be overcome by depleting the glycolysis. An induction of apoptosis by gemcitabine was achieved in the presence of 2DG as depicted by activation of apoptotic protein, Bax, cleavage of PARP and inhibition of antiapoptotic Bcl-2. Our observations identify that modulation of PKD1 expression can turn the PanCa cells sensitive to therapy. This was indicated by using siRNA-mediated knockdown of PKD1 that rendered these cells sensitive to gemcitabine treatment.

In conclusion, we present the expression profile of PKD1 in human PanCa tissues and demonstrate that PKD1 is aberrantly expressed in PanCa as compared with normal pancreatic tissues. Our results suggest a novel role of PKD1 contributing to aberrant glucose metabolism in pancreatic cancer cells, which accelerates PanCa tumorigenesis and chemo-resistance. These interesting findings strongly indicate that PKD1 can be explored as a novel therapeutic target and as a biomarker, inhibition of which can reduce pancreatic cancer progression and metastasis. The association of PKD1-induced mTOR pathway can be used as a therapeutic strategy for the development of new drugs to target pancreatic cancer. Moreover, as suggested by our results, PKD1 has a role in chemo-resistance owing to dysregulated glucose metabolism in pancreatic cancer. Therefore, it can further be exploited to increase the survival rate of pancreatic cancer patients. In conclusion, the inhibition of PKD1 by using pharmacological agents and small-interfering RNA/microRNA-mediated techniques can selectively suppress pancreatic tumour growth and metastasis via inhibition of glycolytic influx in PDAC cells.

## Supplementary information

Supplementary material

Supplementary Figure 1

Supplementary Figure 2

Supplementary Figure 3

Supplementary Figure 4

## Data Availability

The data sets analysed during this study are available from the corresponding author on reasonable request.
