# Supplementary material for: Protein kinase D1 regulates metabolic switch in pancreatic cancer via modulation of mTORC1
Source: Br J Cancer. 2019 Dec 10;122(1):121–31. doi: 10.1038/s41416-019-0629-9 (PMC6964700; doi:10.1038/s41416-019-0629-9)
Supplement: Supplementary file 1 — Supplementary material [file 41416_2019_629_MOESM1_ESM.docx]

**Supplementary figure Legends:**

**Figure S1: Analysis of online datasets for investigating the PKD1 expression in pancreatic cancer.** Two datasets (Segara and Badea) were selected from Oncomine database and analyzed for PKD1 expression in pancreatic cancer and normal human tissues. The dataset representing the log_2_ median-centered intensity for PKD1 expression was used to plot a graph.

**Figure S2: PKD1 overexpression increases cell proliferation in pancreatic cancer cells.** Effect of PKD1 on the morphology of cells (upper panel) and bars representing the cell count acquired using cell countess (Lower panel) in **(A)** HPAF-II and **(B)** BxPC3 cells. Images are captured at 200X magnification.

**Figure S3: Overexpression of PKD1 enhances clonogenic abilities of pancreatic cancer cells under hypoxic environment.** Colony formation assay was performed under normoxic and 1% O_2_ (hypoxic) environment. 250 cells were seeded in 12 wells plate for 7 days and fixed/ stained with crystal violet. Representative images depicting the changes in colony formation of cells in HPAF-II and BxPC-3 cells. **(B)** Bars representing the quantification of the number of colonies. P-values are denoted as *p<0.05.

**Figure S4: Immunoblotting demonstrates the silencing of the Raptor and Rictor expression using siRNA transfection. (A)** Transfection using siRNA directed against Raptor and Rictor confirms silencing of both the proteins by 70-80% in BxPC3 cells as demonstrated by immunoblotting.
