# Supplementary figures and images for: Protein kinase D1 regulates metabolic switch in pancreatic cancer via modulation of mTORC1
Source: Br J Cancer. 2019 Dec 10;122(1):121–31. doi: 10.1038/s41416-019-0629-9 (PMC6964700; doi:10.1038/s41416-019-0629-9)

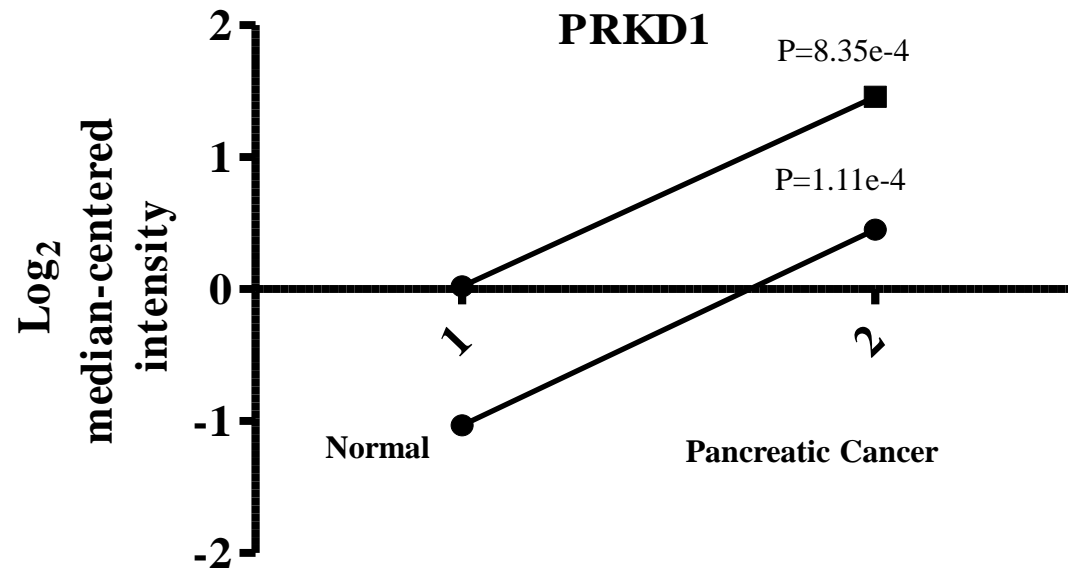

Figure S1

Supplement: Supplementary file 2 — Supplementary Figure 1 [file 41416_2019_629_MOESM2_ESM.pdf]

**A HPAF-II**

**Upper panel**

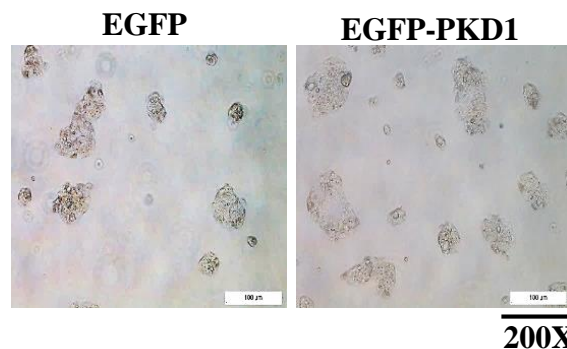

**Lower panel**

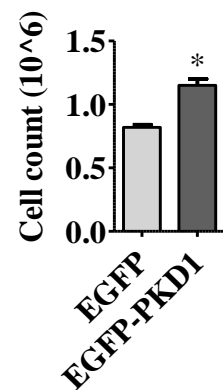

**B BxPC3**

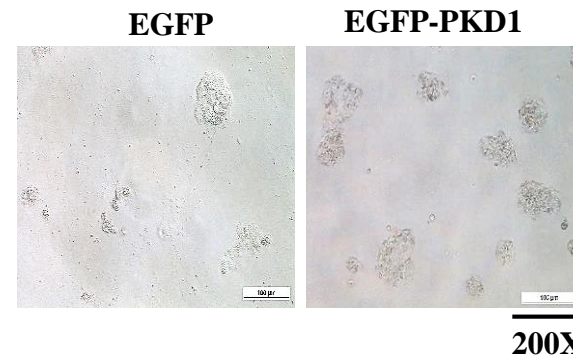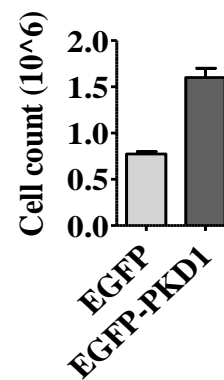

**Figure S2**

Supplement: Supplementary file 3 — Supplementary Figure 2 [file 41416_2019_629_MOESM3_ESM.pdf]

Cells: HPAF-II

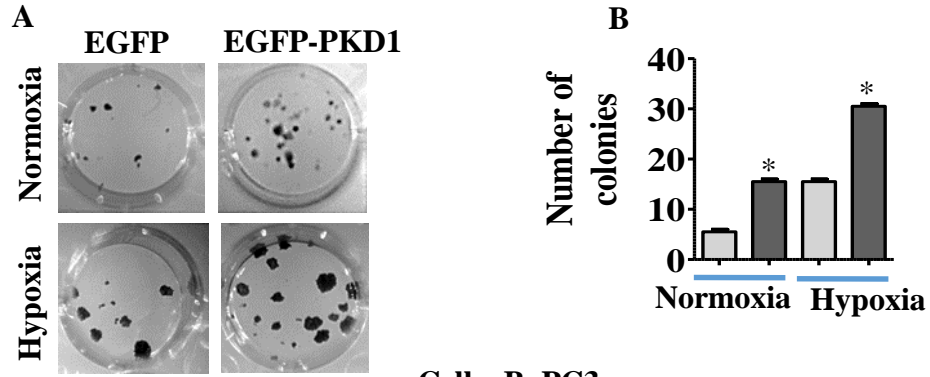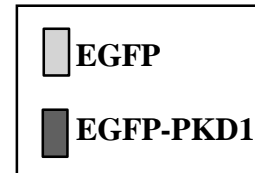

Cells: BxPC3

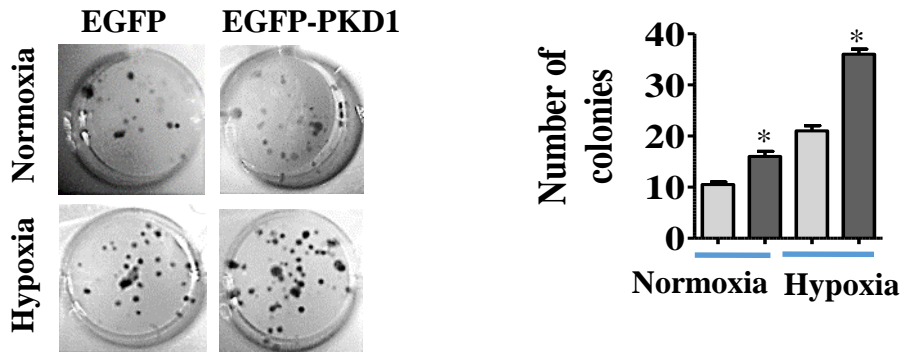

**Figure S3**

Supplement: Supplementary file 4 — Supplementary Figure 3 [file 41416_2019_629_MOESM4_ESM.pdf]

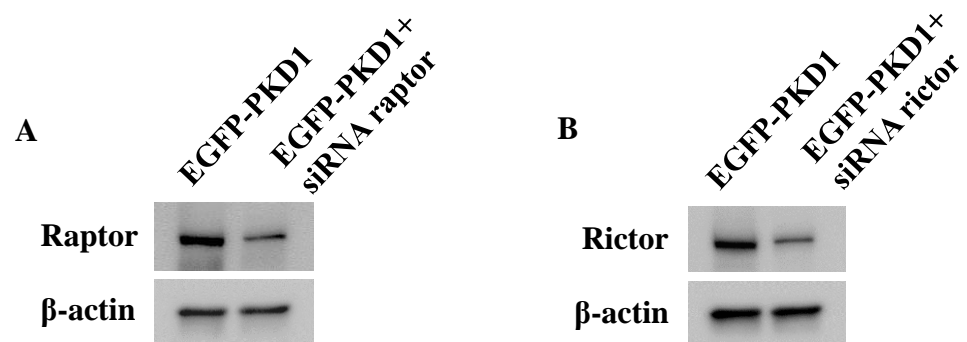

**Figure S4**

Supplement: Supplementary file 5 — Supplementary Figure 4 [file 41416_2019_629_MOESM5_ESM.pdf]
